# Perspectives on the Application of Biosensors for the Early Detection of Oral Cancer

**DOI:** 10.3390/s25051459

**Published:** 2025-02-27

**Authors:** Sanket Naresh Nagdeve, Baviththira Suganthan, Ramaraja P. Ramasamy

**Affiliations:** Nano Electrochemistry Laboratory, School of Chemical, Materials and Biomedical Engineering, University of Georgia, Athens, GA 30602, USA; sanket.nagdeve@uga.edu (S.N.N.); baviththira.suganthan@uga.edu (B.S.)

**Keywords:** biomarkers, biofluids, electrochemical sensors, molecular analytical techniques, diagnostic tools, commercial test kits

## Abstract

Oral cancer continues to cause profound suffering and is associated with high mortality rates. Early detection techniques are crucial in enhancing patient outcomes. This review paper thoroughly evaluates the significance of biomarkers and recent advancements in oral cancer detection, emphasizing cutting-edge electrochemical methods. The paper provides an epidemiological and etiological overview, outlining its clinical importance and reviewing the current state of the art in detection methods. Despite considerable progress, conventional methods exhibit limitations such as invasiveness, long wait times, and a lack of accuracy, creating a critical need for more robust technologies. This review emphasizes the significance of oral cancer biomarkers, which are considered promising cues for early detection, facilitating the development of innovative biosensing technologies. This review seeks to illuminate the recent advances in early detection and precision diagnostics, along with the usage of artificial intelligence strategies, ultimately contributing to significant progress in the battle against oral cancer.

## 1. Introduction: Understanding the Etiology, Prevalence, and Current Diagnostic Tools and Emphasizing the Importance of Early Detection

**Figure 1:**
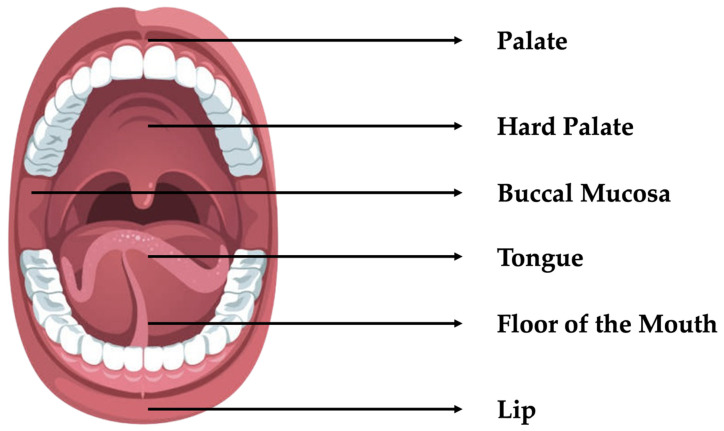
The most common sites of oral cancer.

Oral cancer ranks sixth globally in terms of cancer incidence. Almost 90% of such cancers are oral squamous cell carcinomas (OSCCs) [1]. This is the most prevalent malignant tumor that develops in the oral cavity, starting at the lips and ending at the anterior surface, and is usually lined by squamous cell epithelia with minor salivary gland involvement [2]. The main factors contributing to OSCC are tobacco, betel quid, alcohol consumption, and a sexually transmitted virus called human papillomavirus (HPV) [3]. Typical symptoms include red or white patches on the tongue, ulcers, unusual bleeding, neck swelling, or a lump [4]. Oral cancer affects various areas within the oral cavity, including the lips, tongue (particularly the sides and dorsal surface), floor of the mouth, buccal mucosa (inner lining of cheeks), palate (hard and soft), gums and gingiva, oropharynx (base of the tongue, tonsils, and soft palate), and retromolar trigone (the area behind the last molar teeth on the lower jaw) [5], as shown in Figure 1.

**Figure 2:**
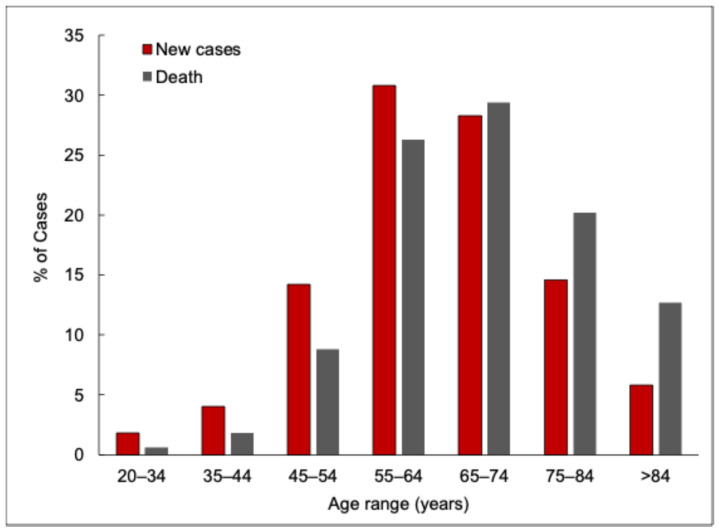
Reported new cases and deaths due to oral cancer across different age groups. (Available online https://seer.cancer.gov/statistics-network/explorer/. Data source(s): SEER Incidence Data, accessed on 20 December 2024) [7].

The Global Cancer Observatory reported 476,000 new cases of oral cancer worldwide in 2020 [6]. Furthermore, data from the National Institute of Dental and Craniofacial Research suggests that the incidence of oral cancer tends to increase with advancing age, particularly among adults aged 65 and older [4]. The five-year survival rate for the period between 2013 and 2019 stood at 68.5%, with approximately 11,580 deaths recorded during this time [7]. Figure 2 showcases the reported new cases and deaths due to oral cancer across different age groups. One of the significant reasons that a large proportion of patients are affected by oral cancer is that it is diagnosed at the later stages.

Studies have reported that oral visual screening can reduce mortality in individuals with significant risk factors (especially tobacco and alcohol users). Visual screening involves thoroughly examining the head, neck, and oral regions to identify premalignant cells [8]. Following this, histopathology also plays an essential role in analyzing the tissue sections by examining the structural and cellular characteristics to determine whether lesions are cancerous or pre-cancerous [9]. Beyond these conventional methods, several adjunctive techniques are also present to enhance oral cancer detection. For example, vital staining is a valuable tool where specific dyes highlight abnormal cells. Similarly, the brush biopsy offers a minimally invasive approach by collecting tissue samples from suspicious areas for detailed analysis. Chemiluminescence or auto-fluorescence is another diagnostic tool that employs a light-sensitive emitting tool to identify malignant tissues for early-stage determination. However, the cornerstone of detection has evolved towards advanced imaging technologies, which play pivotal roles in the precise assessment of malignancies. The magnetic resonance imaging (MRI) technique uses strong magnetic fields that provide extensive and detailed images of the mouth and surrounding tissues to determine the tumor size, location, and extent of invasion [10,11]. Alongside MRI, ultrasound assesses tumor growth in the throat and neck lymph nodes, and computed tomography (CT) provides comprehensive images of oral cross-sectional areas. Other methods, including optical coherence tomography and positron emission tomography, use light or radioactive material to identify cancerous growths throughout the body. Recent advances have introduced several sophisticated approaches, including photoacoustic imaging, which combines ultrasound with optical imaging properties for the staging of cancerous lesions [12,13]. However, these expensive and power-intensive methods have not penetrated the market to become readily available for people with low-income backgrounds. For example, X-rays struggle in soft tissue visualization, while CT scans involve radiation exposure among patients [14,15]. Meanwhile, MRI may not be able to distinguish between benign and malignant lesions consistently, and ultrasound imaging is limited by its penetration depth into the tissue, which compromises its utility in deeper and metastatic lymph nodes [16]. These issues collectively impact the feasibility of and equitable access to diagnostic methods, potentially hindering the cost-effective delivery of dental healthcare services. Raman et al. reviewed several studies on oral and salivary gland cancers, highlighting the effectiveness of oral cancer screening programs. According to the review, the total societal costs depend on several parameters, such as the screening strategies, treatment provided, location, etc. In a community-based program in the United States (US), the cost per screening was estimated at USD 50 to USD 100 per individual, depending on the resources obtained and utilized and the population [17]. Some studies have also reported the quality-adjusted life years (QALY), which vary from USD 20,000 to USD 50,000 in the US [17]. QALY is a measure of the quantity and quality of life used to assess health outcomes, and one QALY is equal to one year of a healthy life. This is usually considered cost-effective in many healthcare systems. However, a limitation of QALY is the availability and quality of the data analyzed. The findings of QALY studies cannot be generalized across different healthcare systems, countries, and populations. Ribeiro-Rotta et al. conducted a comprehensive assessment of the economic burden of oral cancer, with direct costs like medical and non-medical costs and indirect costs like early deaths. Several parameters were identified in the study, including the cost per patient, total cost in a period, and cost per treatment [18]. It also included the inpatient and outpatient costs and the stages of the cancer. It was found that the inpatient costs were 968% higher than the outpatient costs.

Thus, these limitations highlight the necessity of advanced diagnostic technologies and additional methods for improved early screening to enhance the clinical outcomes for patients. From the perspective of the patient, early detection offers profound psychological and physiological benefits, such as identifying malignant lesions at the early stages, reducing the psychological trauma of costly cancer treatment, and increasing the likelihood of complete recovery. Consequently, there is an ongoing demand for more advanced and comprehensive screening strategies. Biomarker-based detection techniques have emerged as a vital early oral cancer diagnosis approach. These molecular methods detect specific indicators of cancer development, potentially offering more precise and comprehensive screening strategies.

**Figure 3:**
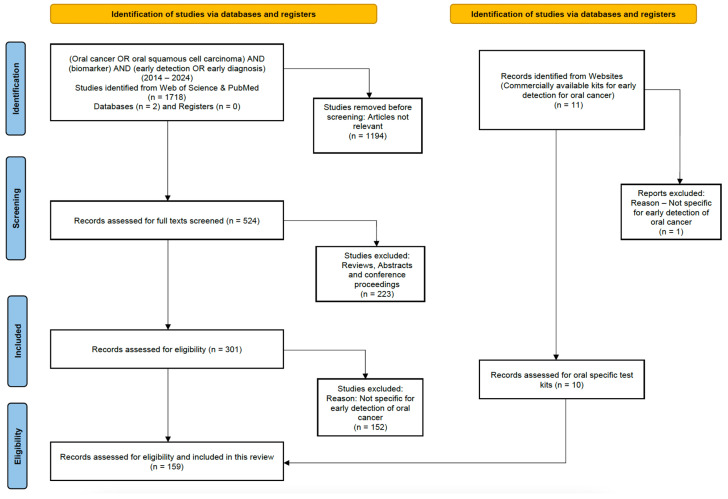
PRISMA flow chart of the study selection process.

The Preferred Reporting Items for Systematic Reviews and Meta-Analyses (PRISMA) flow diagram was used to report the studies involved in this non-meta literature review (Figure 3). A comprehensive search was conducted using keywords on PUBMED, Web of Science, and individual websites using the Google search engine. The keywords used were (“oral cancer” OR “oral squamous cell carcinoma”) AND (“biomarker”) AND (“early detection” OR “early diagnosis”). The studies were searched from 2014 to 2024 and we excluded citations, conference proceedings, abstracts, and any systematic reviews. An additional search was conducted to analyze any commercially available kits for the early detection of oral cancer. The search also focused on studies specifically targeting the early detection or diagnosis of oral cancer biomarkers, with an additional searching criterion of electrochemical biosensors for early biomarker detection. This included the three main electrochemical methods reported in this review (amperometric, voltammetric, and impedimetric). The original research articles included were in the English language, and research papers published before 2014, book chapters, and those in other languages were excluded from this review. To illustrate the usability of this framework, the biomarkers specific to oral cancer were identified, their current molecular detection strategies were studied, and the need for electrochemical biosensors was analyzed. An overview of such biosensors was created, with their fabrication, the strategies involved, and the need for early detection biomarkers associated with oral cancer, as well as their limits of detection (LODs).

## 2. Biomarkers: Significance, Types, and Molecular Detection Strategies

Biomarkers are essential molecular indicators of the normal or malignant functioning of cells and help to determine the diagnosis and prognosis of a particular disease. Biomarkers are valuable in screening individuals without clinical or histological signs of oral cancer, as well as healthy individuals. Their use in early detection significantly reduces patient morbidity and mortality, underscoring the importance of developing clinically validated biomarkers for effective oral cancer screening. Such biomarkers include deoxyribonucleic acid (DNA), microRNAs (miRNAs), long non-coding ribonucleic acids (lncRNAs), messenger RNAs (mRNAs), and proteins. DNA biomarkers involve alterations in the DNA sequence, such as mutations or methylation patterns, associated with oral cancer progression [19], whereas RNA biomarkers play crucial roles in cancer by modulating gene expression [20]. In addition, proteins are associated with various metabolic, structural, and regulatory functions in disease progression [21].

### 2.1. DNA Biomarkers

DNA biomarkers are complex molecules found in every cell of the body. They contain all of the necessary genetic information for the development and functioning of an organism. Gene mutations cause most malignancies, and molecular-based techniques are used to develop and improve diagnostic procedures [22]. Several studies have delved into the characterization of DNA biomarkers related to oral cancer. For instance, the overexpression of NCBP2 and TFRC has been identified in tumor cells, demonstrating significantly higher expression levels than most normal human tissues [23]. On the other hand, emerging DNA methylation markers, such as HOXA1 3′UTR methylation, have been recognized for their potential as predictive biomarkers for OSCC [24]. These findings underscore the critical role of DNA biomarkers in understanding the molecular landscape of oral cancer and their potential applications in diagnostic and predictive settings. The investigation of molecular markers for early-stage OSCC has also been highlighted, emphasizing the need for the comprehensive analysis of such biomarkers to enable optimal clinical treatments and improve patients’ survival rates [25]. These studies collectively advance our understanding of the DNA biomarkers associated with oral cancer, shedding light on their regular and overexpressed levels and their potential significance in clinical practice. However, the performance is not always satisfactory due to DNA denaturation [26]. Although DNA biomarkers provide valuable insights into genetic predispositions and mutations, recently, miRNAs have offered a more nuanced understanding of post-transcriptional gene regulation [20]. Given the dynamic nature of biomarker research, the exploration of miRNAs opens up new avenues for precision medicine and personalized diagnostics, complementing the insights gained from DNA-based analyses. As research has advanced, the focus on biomarker discovery has expanded beyond DNA to explore the potential of miRNAs as novel indicators of biological processes and disease states.

### 2.2. RNA Biomarkers

RNA biomarkers are molecular indicators derived from RNA molecules that provide valuable insights into physiological and pathological conditions within the body. They provide essential information about specific biological conditions. RNA biomarkers are molecular signatures derived from RNA molecules, including mRNA, miRNA, and other non-coding RNAs [27]. RNA molecules play a crucial role in gene expression and regulatory mechanisms. They are used to diagnose diseases and monitor the progression of tumors, as well as the patient’s response to therapy. mRNA biomarkers identify key oncogenes or tumor suppressor genes that are dysregulated in oral cancer [28]. For instance, epidermal growth factor receptor (EGFR) and cyclin D1 can be detected through mRNA analysis and can be correlated with tumor progression or a poor prognosis [29,30]. Similarly, lncRNAs are a group of non-coding RNAs longer than 200 nucleotides that modulate chromatin organization, transcription control, and overall stability. Any aberrant expression in lncRNAs, including HOTAIR and MALAT1, can be considered an early detection biomarker [31,32].

In recent years, miRNAs have emerged as pivotal elements in the pathogenesis of oral cancer, offering potential diagnostic utility and therapeutic value. miRNAs are small non-coding RNAs that govern several physiological processes and have been identified as critical elements in the pathological mechanisms of diverse diseases, including oral cancer [33,34]. These miRNAs are widely distributed in body fluids and demonstrate disease-specific expression patterns, making them potential biomarkers for early detection. Moreover, the inherent stability of miRNAs in bodily fluids, such as serum and saliva, underscores their possible applicability in early cancer diagnosis, highlighting their value as diagnostic biomarkers [33].

Circulating miRNAs, a subset of miRNAs, hold immense promise as diagnostic biomarkers for various types of cancer [35]. Their utility stems from their potential as molecular labels of tumor cells throughout tumorigenesis and cancer progression, with their expression patterns changing as the disease advances. Specific circulating miRNAs, such as miRNA-21, have demonstrated diagnostic potential for certain cancers, offering moderate sensitivity [36]. Notably, a profiling study revealed that miRNAs are differentially expressed in OSCC patients compared to healthy individuals. Therefore, specific miRNAs may serve as promising diagnostic biomarkers, as evidenced by the identification of various miRNAs that show potential for discrimination between oral cancer patients and healthy individuals [33]. Furthermore, specific miRNAs have been associated with clinical staging, metastasis, and overall survival in oral cancer patients [33]. He et al. identified miRNA-24-3p as a potential novel diagnostic salivary biomarker directly associated with the proliferation of cancer cells [37].

Continued research efforts and technological innovations in miRNA-based diagnostic tools are essential in advancing the detection and management of oral cancer, thereby improving patient outcomes and prognoses. miRNAs have exhibited strong potential diagnostic and prognostic value in the context of oral cancer, especially with their distinct expression profiles between healthy individuals and cancer patients and their association with clinical parameters and patient outcomes, underlining their significance as promising biomarkers for early diagnosis and prognostic assessment in oral cancer. As research progresses, it is becoming increasingly evident that miRNAs hold substantial promise, offering novel diagnostic and therapeutic avenues, thus signifying their potential as innovative tools to address the challenges in diagnosing and managing oral cancer. While miRNAs offer valuable information due to their stability and association with disease states [38], protein biomarkers also present a complementary avenue, leveraging proteins’ diverse functions and interactions within biological systems.

### 2.3. Protein Biomarkers

Protein biomarkers play a crucial role in understanding the molecular landscape of oral cancer and its clinical implications. The analysis of the protein expression levels in oral cancer samples reveals the complex divergence between the normal and overexpressed states of specific proteins, providing insights for diagnostic and prognostic evaluations. Protein biomarkers can be detected in various biofluids and are used extensively for disease diagnosis, prognosis, and monitoring. As a promising alternative, oral fluids, such as saliva, human serum, and urine, have gained attention as potential bio-media for oral cancer diagnostics [39].

A large group of protein biomarkers, such as interleukin-6 (IL-6), -8, -1α, and -1β, are commonly used indicators for OSCC diagnosis due to their association with lesion transformation in oral cancer [40]. The use of cytokeratin 19 fragment (CYFRA) 21-1 as a diagnostic biomarker for various cancers, including OSCC, head and neck cancer, bladder cancer, and intrahepatic cholangiocarcinoma, has been explored in several studies. Alali et al. and Liu et al. found that CYFRA 21-1 had high specificity in detecting OSCC and head and neck cancer, respectively, but its sensitivity was low [41,42]. These findings underscore the critical role of protein biomarkers in elucidating oral cancer’s molecular intricacies and their potential as diagnostic and prognostic indicators. By illuminating specific proteins’ normal and overexpressed levels, researchers are paving the way for the development of targeted therapeutic interventions and advancements in personalized medicine in oral cancer management. Huang et al. and Guowei et al. reported the high specificity of CYFRA 21-1 in diagnosing bladder cancer and intrahepatic cholangiocarcinoma, respectively [43,44]. However, the sensitivity of CYFRA 21-1 in these cancers was low. These findings indicate that, while CYFRA 21-1 holds potential as a valuable biomarker in detecting these cancers, its sensitivity and specificity show variability. Therefore, achieving clinical sensitivity and specificity is crucial in detecting protein biomarkers, particularly due to the challenges posed by low sample concentrations and non-specific interactions [45].

Specific proteins, including DEP Domain Containing 1B (DEPDC1B), demonstrate enhanced expression levels in oral cancer samples compared to normal adjacent tissues. This overexpression signifies the potential utility of DEPDC1B as a biomarker for oral cancer [19]. Moreover, proteomic studies have highlighted the differential expression patterns of several proteins in oral cancer. The overexpression of TYPH, an angiogenesis-associated protein, has been identified in many solid tumors, indicating its induction by multiple cytokines and significant contribution to angiogenesis [46]. Conversely, specific proteins in oral cancer samples have shown lower protein expression levels. For instance, the secreted protein acidic and rich in cysteine has shown decreased expression in OSCC tissues, indicating potential alterations in its protective role in the tumor microenvironment [47].

In disease monitoring and detection, biomarkers such as DNA, miRNA, and proteins are essential. DNA biomarkers provide information about the onset and course of the disease. In contrast, miRNA biomarkers control gene expression, and the stability of miRNAs in biofluids is noteworthy, indicating their potential for non-invasive diagnostic applications [35]. On the other hand, specific proteins linked to oral disease can be found in biofluids like saliva, offering important data for prognosis and early detection.

### 2.4. Presence of Biomarkers in Saliva, Serum, and Urine

Oral fluids, including saliva, serum, and urine, are valuable biofluids for the detection of oral cancer due to the presence of specific biomarkers. These biomarkers, including DNA molecules, proteins, and RNA molecules, can indicate the presence of oral cancer and aid in its early detection and monitoring. Salivary biomarkers, for instance, play a crucial role in facilitating the detection and monitoring of oral cancer. Similarly, biomarkers in urine and serum also contribute to effectively detecting oral cancer. Saliva, a biofluid that is abundant in proteins and enzymes, provides a non-invasive means of detecting oral cancer biomarkers. This valuable characteristic makes saliva an essential medium for the early detection and monitoring of oral cancer. Similarly, serum, the precise component of blood without clotting factors, contains specific proteins and molecules that indicate oral cancer, enhancing its utility as a biofluid for diagnostic purposes. Furthermore, despite not originating directly from the oral cavity, urine can offer insights into systemic changes associated with oral cancer, as it harbors metabolites and proteins linked to the disease [48]. These distinct biofluids collectively offer non-invasive and potentially early detection methods, significantly contributing to the management and treatment of oral cancer.

Saliva is a watery extracellular liquid usually secreted by the salivary glands inside the mouth. Whole saliva contains various cellular components, bacteria, viruses, and fungal information, which is critical for any disease analysis. The direct contact of saliva with oral lesions makes it a specific screening tool for the identification of hundreds of cancer biomarkers [49]. The development of screening technologies has shown a promising future for salivary biomarkers in the early detection of oral cancer. Saliva has been extensively studied to discover oral cancer biomarkers [49]. It contains various biomolecules that can indicate the presence of OSCC, offering non-invasive and convenient strategies for diagnosis [40]. Researchers have identified salivary metabolomic biomarkers correlating with oral cancer, aiding early detection [50].

Salivary protein biomarkers for the early diagnosis and prevention of oral cancer include IL-8, IL-6, and tumor necrosis factor α (TNF-α) [51]. Among several groups of proteomic biomarkers in OSCC, interleukins play a vital role in the immune response and the transformation to malignancies [51]. In addition to interleukins, Ricardi et al. identified several critical sources of such biomarkers, including cytokines, growth factors, matrix metalloproteinases (MMPs), acute-phase proteins, and proline-rich proteins [52]. In recent years, saliva-based samples have become more accessible, reliable, and non-invasive. However, the utility of salivary biomarkers in the early diagnosis of OSCC is still under debate, with some studies showing the potential for discrimination between healthy and cancer patients [53].

Serum is another biofluid explored for its potential in oral cancer detection. Proteins, enzymes, hormones, and antibodies are a few of the many biomolecules found in serum, the liquid part of the blood left over after blood clotting [39]. These biomolecules can serve as valuable indicators of various physiological and pathological processes, including the presence and progression of oral cancer. These biomarkers can include tumor-associated proteins, circulating tumor DNA (ctDNA), miRNAs, and other metabolites [39]. Although serum biomarkers hold great promise, further research is needed to establish their clinical utility and reliability for oral cancer detection. While not directly from the oral cavity, serum contains proteins and molecules indicative of oral cancer. Serum contains useful biomarkers that are usually detected by biochemical analysis, providing essential insights into the presence of oral cancer [54].

Urine is another biofluid explored for its potential in oral cancer detection. Although oral cancer primarily affects the oral cavity, specific biomarkers associated with the disease can be detected in urine samples. Urine samples can be collected repeatedly, allowing for longitudinal monitoring and the tracking of changes in biomarker levels [55]. While less commonly studied for oral cancer detection, urine can provide systemic insights into the disease. The metabolites and proteins present in urine can reflect systemic changes associated with oral cancer, offering a potential avenue for diagnostic research [39]. Table 1 highlights the different biomarkers used in the detection of oral cancer.

Among the various biomarkers identified for OSCC, the most prominent ones are determined based on their specificity and utility at different stages of the cancer. Early-stage biomarkers are useful in detecting pre-cancerous lesions. For example, tumor protein 53 (TP53), miRNA-21, EGFR, cyclin D1, and MMPs are observed in oral tissues and are indicative of abnormal cell cycle regulation and extra cellular matrix degradation. The presence of TP53 is one of the earliest molecular events in OSCC progression and plays a major role in regulating cell cycle checkpoints, apoptosis, and DNA repair. Therefore, any TP53 mutation or loss of functionality often indicates pre-cancerous tissue. Similarly, the overexpression of miRNA-21 and cyclin D1 is a hallmark of tumor initiation. Some intermediate and late-stage biomarkers include vascular endothelial growth factor (VEGF), miRNA-10b, IL-6, cancer antigen 125, etc. These late-stage biomarkers are often related to tumor progression, invasion, and metastasis. Their elevated levels in the saliva or serum are linked to increased angiogenesis, disease progression, and localized spread to other body areas. By identifying and understanding the molecular mechanisms of these biomarkers, a critical foundation for early diagnosis and disease monitoring can be leveraged. Building on these insights, the application of advanced molecular techniques enables the precise identification of OSCC biomarkers to determine the required clinical actions.

Molecular and genomic-based approaches have been widely used for oral cancer diagnostics as they are precise, thereby offering the potential for targeted therapies. Similarly, liquid biopsy detects oral squamous cell molecular biomarkers such as ctDNA, miRNAs, lncRNAs, and proteins in bodily fluids like saliva, serum, and blood for the systemic monitoring of the progression of oral lesions. Complementing these advanced techniques, immunoassays provide useful diagnostic information, although they show moderate sensitivity for early-stage detection [12]. Enzymatic and biochemical tests offer a more cost-effective approach to biomarker detection, although they may lack the specificity of newer molecular methods. When combined with imaging and cytological methods, these molecular approaches create a comprehensive diagnostic framework that enhances the accuracy and reliability of oral cancer detection and monitoring.

Among several molecular analytical techniques, nucleic acid-based approaches such as quantitative PCR (qPCR) enable the real-time monitoring and quantification of specific DNA sequences, providing valuable information related to gene mutations, deletions, and amplifications [81]. Such gene alterations are associated with oral cancer; therefore, qPCR offers high sensitivity and specificity. Additionally, quantitative reverse transcriptase polymerase chain reaction (qRT-PCR) is also used for the early detection of gene alterations. It involves multiple steps, such as RNA isolation and reverse transcription [82]. DNA microarrays and next-generation sequencing allow the analysis of genetic mutations by binding DNA or RNA fragments to a solid surface, and the microarray is scanned to measure the expression of each gene printed on the experimental slide [83,84,85]. If a particular gene is different from the reference one, fluorescence is emitted, and the data analyzed provide information that is critical in understanding the presence of oral lesions. However, these techniques are extremely intrusive and cumbersome, require trained personnel, and are time-consuming.

Several user-friendly and non-invasive oral cancer detection kits have been developed recently, providing various screening approaches. Table 2 showcases the features of these commercially available detection kits. Although commercially available kits have been widely used, they are limited by certain characteristics. For example, the commercial kit ViziLite Plus offers insights specifically into HPV-related oral cancer, but non-HPV-related cancer information can be compromised [86]. Similarly, there is a risk of not identifying abnormal cells in mouth areas that are not inspected, and this could lead to lower sensitivity. The precision of such kits also depends on the quality, quantity, and type of the sample collected, which might lead to an inaccurate diagnosis. Some commercially available kits rely on visual inspection methods, either by light-based or dye-based examination. For example, kits such as OralID, Oral Scan Pro, and the Bio/Screen Oral Cancer Screening Kit use fluorescent imaging technologies. This introduces subjectivity, as the interpretation could vary between different examiners with differences in experience and expertise. In addition, a general drawback is false positives due to tissue inflammation or benign lesions rather than actual malignant ones. This can lead to unnecessary follow-up procedures, resulting in greater time consumption and higher expenses. Therefore, additional research is needed to ensure the utmost accuracy and reliability in minimizing the global impact of oral cancer. In this context, biosensors have emerged as novel diagnostic tools for the detection of oral carcinoma, seeking to investigate the biomarkers responsible for oral cancer malignancies and to diagnose it in the early stages.

## 3. Advancing Biomarker Detection with Electrochemical Biosensors

Biosensors are diagnostic tools that offer compelling advantages over conventional practices. By harnessing biological interactions, biosensors provide non-invasive, rapid, and cost-effective diagnostic solutions to improve oral healthcare standards. They are suitable due to their fast responses, reliability, and direct sample reading, without needing to rely on a laboratory for analysis. Their advantages also include portability, simplicity, sensitivity, a small size, low costs, rapidity, ease of use, and wide range of concentrations [96]. They comprise a biorecognition probe for the specific target analyte of interest, a transducer, and a signal processing system integrated into a device or assembly. Within biosensors, the biorecognition probe encompasses various components, such as proteins (natural or engineered antibodies, enzymes, plant proteins), whole cells (bacteria), and nucleic acids (DNA, RNA, aptamers), which are immobilized on a transducer platform [97]. The biological signal obtained from the interaction between the analyte and biorecognition molecule is transformed and measured in electrical, fluorometric, luminometric, or colorimetric signals, confirming the presence or absence of the bio-analytes of interest [98]. These bio-analytes are commonly found in biological fluids, including the blood, serum, urine, saliva, and other bodily fluids, and they serve as essential targets for biosensor detection [99]. Utilizing these bio-analytes as targets (analytes) for biosensing applications provides a rapid and selective analysis, which is crucial for medical diagnostics and pharmaceutical development.

Within the realm of biosensors, various biosensing platforms have been developed, including lateral flow assays (LFAs) and colorimetric, optical, and electrochemical biosensors. LFAs are portable devices that exhibit a color change response upon detecting the target analyte, characterized by their simplicity, cost-effectiveness, and rapid testing capabilities in identifying biomarkers in bodily fluids. They require minimal sample preparation and usually provide results within minutes. This makes LFAs valuable for early screening, without the need for complex instruments. However, they can pose a challenge in low concentrations. Recent studies have highlighted the application of LFAs using microfluidic-based colorimetric approaches with point-of-care devices for oral cancer [100]. Similarly, colorimetric biosensors are based on the principle of a visible color change when a reaction between the bioreceptor and the analyte occurs. An immediate color change indicates the presence of a biomarker; however, the color change could also be affected by complicated samples or environmental changes [22,101]. Likewise, optical biosensors detect changes in light properties during a biological interaction, thereby being useful for the real-time monitoring of biological reactive systems [102]. These techniques may include fluorescence and surface plasmon resonance, which can function in label-free modes; however, they require sophisticated equipment, such as lasers and photodetectors, which may not be appropriate in terms of portability.

On the other hand, electrochemical biosensors record and monitor changes in electrical signals due to electrochemical reactions between the target molecules and the biorecognition molecules (enzymes, antibodies, and synthetic molecules like aptamers, DNA fragments, peptides, etc.) [103]. Electrochemical biosensors can efficiently detect biomarkers in body fluids, such as sweat, blood, or feces, making them vital for health monitoring, disease diagnosis, and environmental analysis [104,105,106]. Understanding the principles and capabilities of such biosensors sets the stage for the exploration of their specific use in oral cancer detection.

In electrochemical biosensors, an electrode, known as a working electrode, serves as a platform for the immobilization of biomolecules and electron mobility, in addition to a counter and reference electrode. This working electrode is the primary electrode where the target interacts with the biorecognition element and is typically modified with nanomaterials and crosslinkers to increase the surface area and conductivity and ensure efficient immobilization [107]. The counter electrode completes the necessary circuit to sustain the current generated at the working electrode, whereas the reference electrode maintains a known and constant potential at the working electrode. Usually, a glassy carbon electrode, a gold electrode, a screen-printed electrode (SPE), or indium tin oxide (ITO) glass is used as the working electrode in the electrochemical setup. Nanomaterials such as graphene, gold nanoparticles, carbon nanotubes, etc., are deposited onto the electrode to increase electron transfer and amplify signals [108]. The immobilization of biomolecules on the electrode is achieved using molecular crosslinkers like carbodiimide 1-ethyl-3-(3-dimethylaminopropyl) carbodiimide (EDC)/N-hydroxysuccinimide (NHS), glutaraldehyde, 1-pyrenebutanoic acid, succinimidyl ester, (3-aminopropyl)triethoxysilane (APTES), etc. [109]. These crosslinkers secure the molecules onto the platform and maintain biological activity for target detection, which is related to the biosensor’s sensitivity and LOD. In terms of electrochemical strategies, several techniques are typically chosen for biosensor development, including cyclic voltammetry (CV), differential pulse voltammetry (DPV), square wave voltammetry (SWV), amperometry, and electrochemical impedance spectroscopy (EIS) [110].

### 3.1. Amperometric Biosensors

Amperometric biosensors function by primarily measuring the current generated by a redox process at a fixed voltage. Such biosensors include an electrode surface that facilitates electron transfer reactions. Upon binding with the target analyte, the recognition element triggers an electrochemical reaction, resulting in a measurable current. The concentration of the desired analyte within the sample is directly correlated with the amplitude of the current. A typical electrochemical technique performed is chronoamperometry, where a constant potential is applied, and the resulting current is measured as a function of time. This change in current is proportional to the amount of redox species that are oxidized and reduced. The capability of amperometric biosensors to enable the reliable and non-invasive detection of biomarkers presents a transformative opportunity for early intervention and enhanced patient outcomes [111].

**Figure 4:**
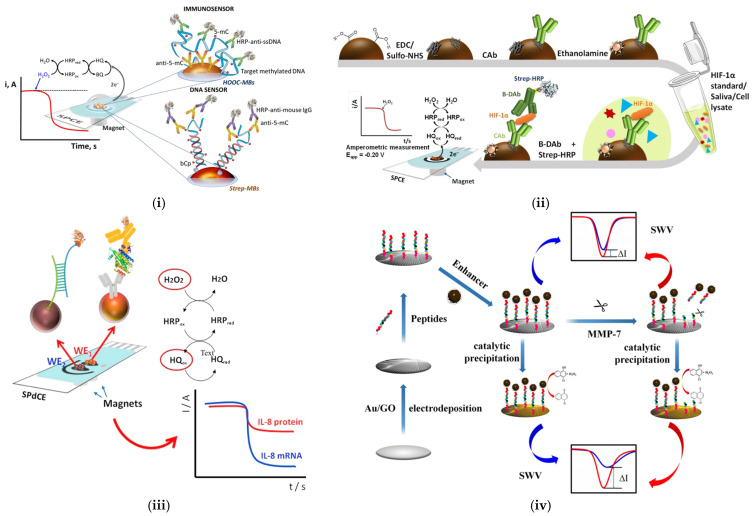
Amperometric biosensors using different immobilization strategies for detection of various oral cancer biomarkers. Schematic representation of the fabrication procedure for (**i**) 5–mC Reprinted with permission from [112]; (**ii**) HIF–1α Reprinted with permission from [113]; (**iii**) IL–8 protein and IL–8 mRNA Reprinted with permission from [114]; (**iv**) MMP–7 Reprinted with permission from [116].

Amperometric biosensors provide a fast and cost-effective solution for the detection of proteins and DNA species associated with oral cancer [111]. Two gene-specific strategies (one DNA sensor and an immunosensor) were employed by Povedano et al. for the development of electrochemical biosensors for the quick detection of DNA methylation. They used functionalized MBs, attaching 5-methylcytosine (mC) as a bio-receptor and achieving amperometric detection by using a hydrogen peroxide/hydroquinone (H_2_O_2_/HQ) system (Figure 4(i)) [112]. The aberrant methylation of DNA is usually observed in tumor cells, which helps in the promotor regions of tumor suppressor genes. Therefore, the inactivation of tumor suppressor genes has been shown to be an important mechanism in the development of human oral cancer.

Hypoxia-inducible factor-1 alpha (HIF-1α) is a transcription factor that plays a role in oral tumor growth and metastasis by regulating genes associated with the cellular response to hypoxia. Martin et al. developed an immunosensing amperometric detection tool to analyze HIF-1α on disposable screen-printed electrodes (Figure 4(ii)) [113]. Magnetic immunoconjugates of carboxylic acid-modified magnetic particles (HOOC-MBs) were utilized to selectively capture the target protein with an EDC/NHS crosslinking mechanism. The biosensor showed great selectivity and a low LOD of 76 pg/mL. This was followed by the determination of HIF-1α in raw saliva samples. Further research in more sensitive electrochemical sensors has paved the way for spatially resolved biosensing and imaging applications, indicating the potential of these biosensors for point-of-care testing in the diagnosis of various diseases. In another study, Torrente-Rodriquez et al. reported the simultaneous detection of IL-8 mRNA and IL-8 protein oral cancer biomarkers in undiluted human saliva using functionalized MBs (Figure 4(iii)) [114]. The use of MBs proves useful for improving the sensitivity and minimizing matrix effects, which makes them a relevant tool in the design of biosensors for complex samples.

Recent advances in amperometric biosensors for medical diagnostics have significantly expanded their applicability in disease detection. The fabrication of amperometric biosensors for the detection of MMP-7 has seen significant advancements, particularly with the use of palladium (Pd)-functionalized carbon nanocomposites as another biomarker. Palomar et al. studied the detection of MMP-7 by integrating peptide-decorated gold nanoparticles and carbon nanotubes, achieving an LOD of 6 pg/mL [115]. Wei et al. further improved the detection limit using a Pd-functionalized carbon nanocomposite, as shown in Figure 4(iv) [116]. A glassy carbon electrode was used to immobilize peptides on gold-modified reduced graphene oxide (rGO), which improved the biosensor’s conductivity and provided more reaction sites for immobilization. Catalytic precipitation with Pd-functionalized carbon nanospheres amplified the signal obtained, resulting in an LOD of 17.38 fg/mL [116]. Table 3 highlights the recent developments in amperometric-based biosensors for the detection of oral cancer biomarkers.

**Table 3:** Amperometric biosensors and their experimental conditions for oral cancer.

Electrode/Platform	Biomarker	Analyte	Method	Detection Limit	Normal and Cancer Patient Levels	Reference
SPCE/Strep–MBs	5–mC	Human saliva, serum, and urine	Amperometry	0.3–1 fg/mL	-	[112]
SPCE/HOOC–MBs	HIF–1α	Human saliva	Amperometry	76 pg/mL	-	[113]
SPE/HOOC–MBs	IL–8 protein	Human saliva	Amperometry	72.4 pg/mL	IL–8 protein—patient: 720 pg/mL, normal: 250 pg/mL	[114]
GCE/Au–rGO	MMP–7	Human serum	Amperometry	1 ng/mL	-	[116]

Abbreviations: SPE—screen-printed electrode, HOOC—carboxylic acid, MBs—magnetic beads, SPCE—screen-printed carbon electrode, Strep-MBs—streptococcal magnetic beads, GCE—glassy carbon electrode, Au—gold, rGO—reduced graphene oxide, IL-8—interleukin-8, HIF-1α—hypoxia-inducible factor-1 alpha, 5-mC—5-methylated cytokine, MMP-7—matrix metalloproteinase-7.

Despite demonstrating significant advancements in the detection of oral cancer, amperometric biosensors possess certain limitations that need to be considered. Signal reduction may occur due to fouling agents and other interfering substances, which poses challenges in maintaining consistent and reliable detection [117]. Furthermore, some amperometric biosensors may have poor specificity, limiting their effectiveness in specific applications [118]. Another aspect of their use that must be considered is the possibility of cross-sensitivity to other substances. These issues emphasize the importance of calibrating the instruments precisely and considering possible interferences while performing detection. A bottleneck in the translation of such biosensors from the lab to industry lies in the stability and activity of the species involved in the electrochemical reaction. Additional efforts are required to address the effects of the temperature, buffer, and pH and the appropriate potential to maintain the stability of the system. Overall, careful calibration that considers the real environmental conditions and potential interferences is essential in accurately applying amperometric biosensors, especially in complex samples such as those for oral cancer detection.

### 3.2. Voltammetric Biosensors

**Figure 5:**
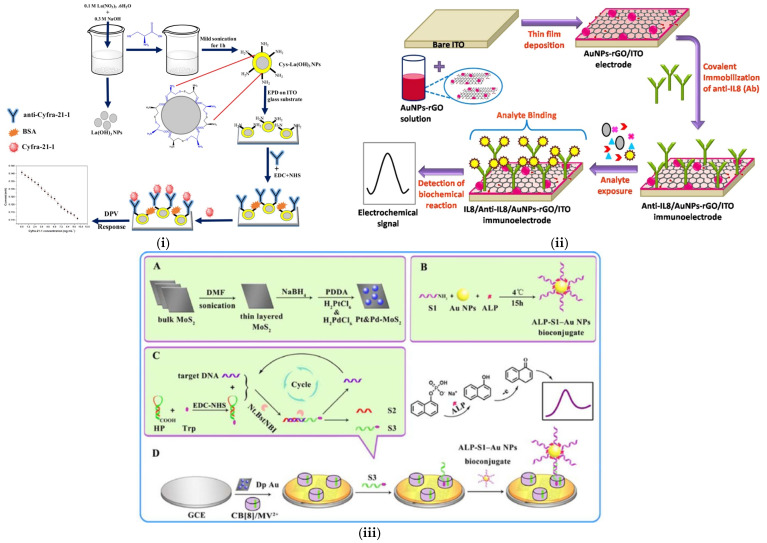
Voltammetric biosensors using different immobilization strategies for detection of various oral cancer biomarkers. Schematic representation of the fabrication procedure for (**i**) CYFRA–21–1 Reprinted with permission from [119]; (**ii**) IL–8 Reprinted with permission from [124]; (**iii**) ORAOV1: (**A**–**C**) represent the preparation steps for composite nanomaterial/bioconjugates and target amplification, while (**D**) shows the preparation process of the biosensor. Reprinted with permission from [128].

Voltammetric biosensors are a class of electrochemical sensors that have gained significant attention in medical diagnostics. Voltammetric biosensors measure the current when the potential is applied at the working electrode, enabling the detection and quantification of target analytes. This binding event between the biorecognition element and the target analyte triggers an oxidation/reduction reaction that forms the basis of electrochemical detection. Several electrochemical techniques are employed, such as CV, DPV, and SWV, to analyze this redox behavior. In CV, the potential of the working electrode is swept linearly and reversed, which generates a current vs. voltage graph, providing the relevant information about the reaction that occurs at the electrode surface. In the case of DPV, small potential pulses are applied to enhance the signal-to-noise ratio, which improves the detection limits, while small steps are applied in SWV. The current generated in such biosensors is directly related to the concentration of the analyte. For example, Tiwari et al. developed a biosensor based on the CV and DPV methods to detect CYFRA-21-1 by immobilizing anti-CYFRA-21-1 antibodies on an ITO platform, as shown in Figure 5(i) [119]. Lanthanum hydroxide (La(OH)_3_) has several electrochemical properties, including electron transfer mobility, which provides more free binding and adsorption sites for the attachment of molecules like L-cysteine. This increases the loading of the anti-CYFRA-21-1 antibodies, which enhances the stability of the electrode. The immunosensor showed a low detection limit of 0.001 ng/mL, with a response time of 5 min. This immunosensor has high sensitivity compared to other commercially available ELISA kits. In a similar context, Jafari et al. immobilized anti-CYFRA-21-1 antibodies on a gold electrode using a cysteamine and glutaraldehyde self-assembly composite [120]. This composite biosensor resulted in a low limit of quantitation of 2.5 ng/mL when using CV and SWV. Because of its quick, reliable, and user-friendly nature, the fabricated layer, with a high current intensity, can be used to monitor oral abnormalities. However, the detection limit is 100 times lower than that of the La(OH_3_)-based nanomaterial reported, and it demonstrates a larger surface area, better redox reactivity, and excellent electrochemical behavior. However, given the need for a low-cost biosensor, La(OH)_3_ could prove to be too expensive, since lanthanum is a rare earth metal. In this case, the biosensor fabricated by Jafari et al. could be useful.

Kumar et al. developed a biocompatible serine-functionalized nanostructured zirconia platform that showed a low detection limit for the oral cancer biomarker CYFRA-21-1 [121]. This biosensing platform was fabricated with nanostructured zirconia with rGO, which exhibited improved electron transfer kinetics and increased sensitivity [121]. In 2015, the same group utilized silanized nanostructured zirconia for the covalent immobilization of monoclonal anti-CYFRA-21-1 antibodies, resulting in a valuable and stable biosensing platform with a lower detection limit of 0.001 ng/mL [122]. More recently, Kumar et al. synthesized nanodot zirconia and developed an efficient biosensing platform with a broad linear detection range and excellent sensitivity for CYFRA-21-1 [123]. However, the LOD was lower as compared to that of the silanized nanostructured zirconia. This indicates that the zirconia surface, when functionalized with silane coupling agents, provides stronger adhesion to the biomolecules, thereby achieving higher surface reactivity and, hence, a lower limit of detection.

The label-free electrochemical detection of oral cancer-related biomarkers has been a recent research focus. Verma et al. demonstrated the fabrication of an electrochemical immunosensor to detect the salivary oral cancer biomarker IL-8 using a gold nanoparticle–rGO composite material (Figure 5(ii)) [124]. The synergy between the gold nanoparticles and rGO led to a faster response and higher sensitivity due to the enhanced electron transfer behavior of the nanocomposite, resulting in a detection limit of 72 pg/mL, as well as excellent specificity in human saliva samples. Liu et al. further advanced this work by creating an MMP-1 immunosensor based on a gold nanoparticle/polyethyleneimine/rGO nanocomposite [125]. Braiek et al. expanded the application of these nanocomposites to the detection of IL-8 using a boron-doped diamond electrode modified with magnetic nanocomposites [126]. These studies collectively demonstrate the potential of several nanocomposites for the sensitive and specific detection of salivary oral cancer biomarkers.

Another mechanism involved in electrochemical biosensors for oral cancer-based DNA detection is based on nicking endonuclease signal amplification (NESA). This is a technique used to detect specific DNA sequences by utilizing an enzyme that only cuts one strand of the DNA to generate a signal that can detected. An NESA-based approach on an ITO electrode was reported by Tan et al. for the detection of DNA to target ORAOV1 in saliva [127]. The DPV responses from the system were detected to evaluate the sensitivity and dynamic range of the biosensor, resulting in an LOD of 0.35 pM. Regarding similar NESA approaches, a sensitive biosensor was successfully constructed by Hu et al. with a novel host–guest recognition system consisting of tryptophan-linked DNA, methyl viologen (MV2+), and cucurbit uril for the sensitive detection of oral cancer genes, as shown in Figure 5(iii) [128]. Platinum (Pt)- and Pd-based nanomaterials were used to detect ORAOV1 using hairpin DNA labeled with tryptophan. The catalysis of the Pt and Pd–molybdenum disulfide composite was not only used to promote electron transfer but also for signal amplification, and the biosensor was reported as a promising tool for ORAOV1 detection on a glassy carbon electrode. Table 4 highlights the recent developments in voltammetric-based biosensors for the detection of oral cancer biomarkers.

One of the key advantages of voltammetric-based electrochemical biosensors is their high sensitivity, which enables the detection of oral cancer biomarkers at low concentrations [129]. Furthermore, these biosensors are known for their rapid detection capabilities, cost-effectiveness, and low production costs, making them accessible for widespread use [130]. However, voltammetric biosensors face limitations in complex oral environments, as well as having a restricted dynamic range and reproducibility [22]. These biosensors utilize electrochemical amplification, surface modifications, nanomaterial integration, specific recognition elements, and sophisticated signal processing to achieve exceptional sensitivity, even at trace biomarker levels. However, the complex oral environment presents challenges to the stability and robustness of voltammetric biosensors, necessitating the use of protective coatings, surface modifications, advanced materials, fluid handling systems, calibration and referencing, optimized recognition elements, environmental controls, signal processing techniques, robust design, and routine maintenance. By implementing these strategies, the reliability and performance of voltammetric biosensors can be enhanced in detecting oral cancer biomarkers, thereby supporting their potential application in early diagnosis and effective treatment management.

**Table 4:** Voltammetric biosensors and their experimental conditions for oral cancer.

Electrode/Platform	Biomarker	Analyte	Method	Detection Limit	Normal and Cancer Patient Levels	Reference
ITO/APTES/ndZrO_2_	CYFRA–21–1	Human saliva	CV/DPV	0.5 ng/mL	Normal—3.3 ng/mL CYFRA–21–1—17.46 ± 1.46 ng/mL	[123]
ITO/L–Cys–La (OH)_3_	CYFRA21–1–1	Human saliva	CV/DPV	0.001 ng/mL	Normal—3.3 ng/mL CYFRA–21–1—17.46 ± 1.46 ng/mL	[119]
ITO/Serine/nZrO_2_	CYFRA–21–1	Human saliva	CV/DPV	0.01 ng/mL	Normal—3.3 ng/mL CYFRA–21–1—17.46 ± 1.46 ng/mL	[121]
ITO/APTES/ZrO_2_ rGO	CYFRA–21–1	Human saliva	DPV	0.122 ng/mL	Normal—3.3 ng/mL CYFRA–21–1—17.46 ± 1.46 ng/mL	[131]
GE/Cys A	CYFRA–21–1	Human saliva	CV/DPV/SWV	2.5 ng/mL	Normal—3.3 ng/mL CYFRA–21–1—17.46 ± 1.46 ng/mL	[120]
ITO/ZnO–rGO	IL–8 protein	Human saliva	CV/DPV	51.53 ± 0.43 pg/mL	Normal—250 pg/mL IL–8 protein, patient—720 pg/mL	[132]
ITO/AuNPs–rGO	IL–8	Human saliva	CV/DPV	72.73 ± 0.18 pg/mL	Normal—250 pg/mL IL–8 protein, patient—720 pg/mL	[124]
GCE/Au/Pt and Pd–MoS_2_	ORAOV1	Artificial saliva	CV/DPV	3 fg/mL	-	[128]
ITO/eMB/Nt.BstNBI	ORAOV1	Human saliva	DPV	0.35 pg/mL	-	[127]

Abbreviations: ITO—indium tin oxide, ZnO—zinc oxide, rGO—reduced graphene oxide, GE—gold electrode, Cys-A—cysteine-A, AuNPs—gold nanoparticles, APTES—(3-aminopropyl) triethoxysilane, ZrO_2_—zirconium oxide, Cys—cysteine, La (OH)_3_—lanthanum hydroxide, GO—graphene oxide, GCE—glassy carbon electrode, nZrO_2_—zirconium oxide, Au—gold, Pt and Pd-MoS_2_—platinum and palladium molybdenum disulfide, eMB—methylene blue, Nt.BstNBI—nicking enzyme, L-Cys—L-cysteine, ndZrO_2_—nanodiamonds zirconium oxide, IL-8—interleukin-8, CYFRA-21-1—cytokeratin 19 fragment, ORAOV1—oral cancer overexpressed 1, CV—cyclic voltammetry, DPV—differential pulse voltammetry, SWV—square wave voltammetry.

### 3.3. Impedimetric Biosensors

**Figure 6:**
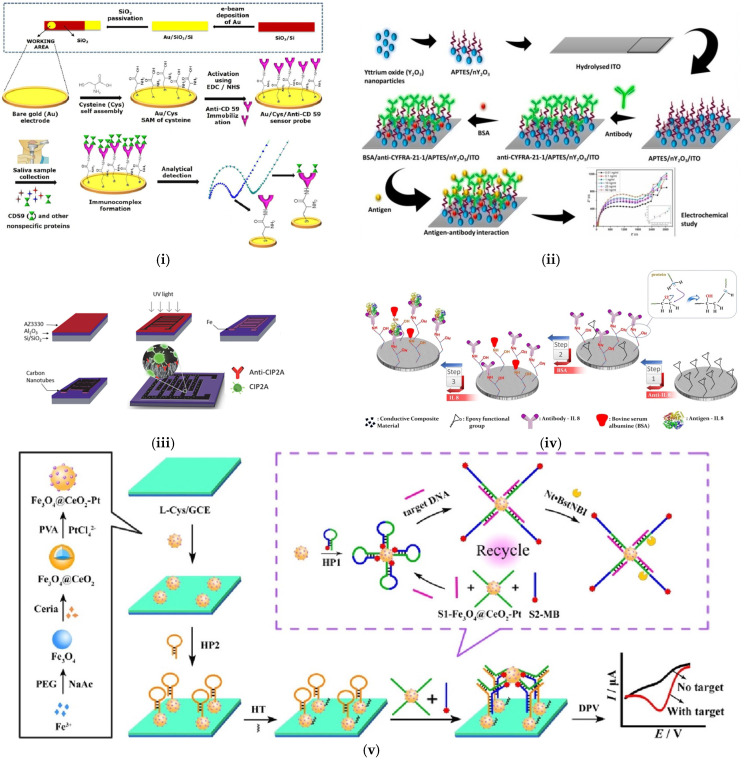
Impedimetric biosensors using different immobilization strategies for detection of various oral cancer biomarkers. Schematic representation of the fabrication procedure for (**i**) CD59 Reprinted with permission from [134]. (**ii**) CYFRA–21–1 Reprinted with permission from [135]; (**iii**) CIP2A Reprinted with permission from [137]. (**iv**) IL–8 Reprinted with permission from [138]; (**v**) ORAOV1 (DNA) Reprinted with permission from [139].

Impedimetric biosensors are advantageous for point-of-care diagnostics because they can identify biorecognition events by measuring the changes in the electrical properties at the electrode/electrolyte interface over a range of frequencies. In impedimetric biosensors, the activity of the target analyte is proportional to the output of an electrical impedance, and a common reporting method is EIS. It can easily help to determine the properties of the processes in the bulk, as well as at the electrode interface, which are represented in the form of a Nyquist plot or Bode plot as a function of the frequency. A Nyquist plot generally consists of a semi-circular region and a straight line that represent the charge transfer process and the diffusion process, respectively, whereas a Bode plot consists of a logarithm of the absolute values of the impedance and phase plotted against the logarithm of the frequency [133]. These plots are used to understand the electrochemical processes involved in the biosensing mechanism, such as charge transfer, electroactive species adsorption, mass transfer, and electrolyte resistance, each represented by an equivalent electrical circuit. In a Nyquist plot, the change in impedance obtained indicates when the receptor binds to the target analyte, and it is proportional to the diameter of the semi-circle. This measurement does not depend on the redox species present in the electrolyte. In order to maintain stability and repeatability, nanomaterials functionalized with different crosslinkers are critical in attaching the relevant receptors. For example, Choudhary et al. developed a highly sensitive and selective label-free impedimetric biosensor on a gold electrode by detecting CD59 as an oral cancer biomarker (Figure 6(i)) [134]. CD59 is a serine protease and a complement restriction factor that can be used as a relevant early-stage biomarker for oral cancer diagnosis. The electrode was functionalized with an L-cysteine self-assembled monolayer to provide carboxyl functional groups for the attachment of CD59 antibodies using EDC-NHS functionalization. EDC-NHS was used as a coupling agent to immobilize the anti-CD59 antibodies by forming covalent bonds between the cysteine-functionalized -COOH groups and the -NH2 groups on the antibodies. This resulted in an LOD of 0.38 fg/mL and 1.46 fg/mL in standard buffer and untreated human saliva samples, respectively [134].

On the other hand, Kumar et al. explored and reported the detection of the CYFRA-21-1 biomarker, as shown in Figure 6(ii) [135]. Herein, the researchers used yttrium oxide (nY_2_O_3_) nanoparticles functionalized using APTES to immobilize anti-CYFRA-21-1 antibodies, followed by a blocking agent [135]. nY_2_O_3_ is known to have a high quantum yield, which creates a highly conductive thin film, thereby making it a potential candidate for the development of biosensors. It resulted in an LOD of 0.33 ng/mL. Similarly, the studies by Pachauri et al. presented innovative biosensing platforms for CYFRA-21-1, using a nanocomposite of ncCeO_2_-rGO (cerium oxide nanocubes–rGO) [136]. The use of ncCeO_2_-rGO provided a larger surface area and faster electron transfer due to the conducting nature of rGO. This also resulted in a better LOD of 0.625 pg/mL as compared to Kumar et al.’s work. However, such studies require a more critical analysis of the potential limitations and challenges in translating these platforms to clinical use. Additionally, the specificity of the biosensors to CYFRA-21-1 in the presence of other biomolecules, such as those found in saliva, needs to be thoroughly addressed.

In addition to CD59 and CYFRA-21-1, several other biomarkers have been used for the electrochemical detection of OSCC. For example, Cancerous Inhibitor of Protein Phosphatase 2A (CIP2A) is highly expressed in OSCC cell lines, in addition to lung and breast cancers. Figure 6(iii) highlights the fabrication steps for such a biosensor, where vertically aligned carbon nanotube (VACNT) array electrodes were used for the electrochemical detection of CIP2A [137]. They had a large surface area that favored electrical conductivity and reactivity, leading to higher biosensor sensitivity compared to the ELISA test. The target CIP2A was detected using CIP2A antibodies (anti-CIP2A) attached to interdigitated electrodes, which acted as electrochemical transducers. Although this biosensor achieved detection in the range of pg/mL, scaling up and maintaining the perfect alignment of VACNT arrays can be expensive and challenging. This is especially important when less expensive nanomaterials, such as graphene or cysteine, are available and offer a similar detection range.

The development of impedimetric biosensors also leverages advanced functional polymers, which play a critical role in the sensitivity and biocompatibility of oral cancer biomarkers. The studies by Aydin et al. present the development of an immunosensor for the detection of IL-8 using a variety of conjugated polymers containing epoxy side groups; see Figure 6(iv) [138]. This study demonstrates the successful immobilization of anti-IL-8 receptors on modified disposable ITO electrodes bound to the epoxy groups of star polymers via a covalent bond. The star polymers provide a convenient interface due to the presence of linear polymer chains in a branched fashion, and the system exhibits remarkable features like a low cost and good feasibility. It resulted in a detection limit of 3.3 fg/mL. Interestingly, Ma et al. developed a ratiometric electrochemical DNA biosensor for ORAOV1, with a detection limit of 12 fM in artificial saliva samples, as shown in Figure 6(v) [139]. The DNA is hybridized with a ferrocene-labeled hairpin probe and separated by exonuclease III, which then releases the target and triggers an amplification cycle, whereas the remaining probe is used for biosensing purposes. The larger the amount of the ORAOV1 DNA biomarker present in the saliva sample, the higher the response detected and, hence, the larger the impedance on the Nyquist plot. These biosensors exhibited a linear calibration curve over a wide concentration range; however, the authors did not critically analyze the potential for non-specific binding or interference from other biomarkers. Additionally, the comparison with commercial ELISA kits was limited to the detection limits and analysis costs, without considering other important factors such as the accuracy and precision. Further research is needed to address these limitations and confirm the clinical utility of these biosensors.

The capacity of impedimetric biosensors to detect the presence of cancer biomarkers makes them precise and sensitive instruments that are essential in the early identification of oral cancer. These biosensors can evaluate biomarkers without labeling the target molecules, simplifying sample preparation and preserving the integrity of biological samples while enhancing their non-invasiveness. In clinical settings, real-time analysis capabilities enable prompt diagnostic feedback, essential for rapid decision-making. Additionally, the small sample size required for impedimetric biosensor testing minimizes the risk of side effects during blood draws and improves patients’ comfort. Table 5 highlights the recent developments in impedimetric-based biosensors for the detection of oral cancer biomarkers.

**Table 5:** Impedimetric biosensors and their experimental conditions for oral cancer.

Electrode/Platform	Biomarker	Analyte	Method	Detection Limit	Normal and Cancer Patient Levels	Reference
GE/Cys	CD59	Human saliva	CV/EIS	Treated saliva: 0.84 ± 0.04 fg/mL; raw saliva: 1.46 ± 0.05 fg/mL	Normal—7.8 ng/mL CD59 patients—27.3 ng/mL	[134]
Silicon oxide wafer/Al_2_O_3_	CIP2A	Human saliva	CV/EIS	0.24 pg/ml	-	[137]
ITO/ncCeO_2_–rGO	CYFRA–21–1	Human saliva	DPV/EIS	0.625 pg/mL	Normal—3.3 ng/mL CYFRA–21–1—17.46 ± 1.46 ng/mL	[136]
ITO/APTES/nY_2_O_3_	CYFRA–21–1	Human saliva	EIS	0.01 ng/mL	Normal—3.3 ng/mL CYFRA–21–1—17.46 ± 1.46 ng/mL	[135]
ITO–PET	IL–8	Human serum and saliva	CV/EIS	3.3 fg/mL	IL–8 protein, patient: 720 pg/mL; normal: 250 pg/mL	[138]
GE/MB–PP1–MCH	ORAOV1	Artificial saliva	EIS	12.8 fg/mL	-	[139]

Abbreviations: ITO—indium tin oxide, PET—polyethylene terephthalate, ncCeO_2_—nanocubes cerium oxide, rGO—reduced graphene oxide, Al_2_O_3_—aluminum oxide, GE—gold electrode, MB-PP1—methylene blue-labeled hairpin probe, MCH—6-mercapto-1-hexanol, Cys—cysteine, APTES—(3-aminopropyl) triethoxysilane, nY_2_O_3_—yttrium oxide, IL-8—interleukin-8, CYFRA-21-1—cytokeratin 19 fragment, CIP2A—Cancerous Inhibitor of Protein Phosphatase 2A, ORAOV1—oral cancer overexpressed 1, CD59—cluster of differentiation 59, CV—cyclic voltammetry, EIS—electrochemical impedance spectroscopy, DPV—differential pulse voltammetry.

Nonetheless, electrochemical biosensors’ practical applications could be improved by addressing the issue of the non-specific binding of non-target compounds, resulting in reduced sensitivity and selectivity. The limitations also include linearity, stability, and the precision of the instruments utilized. Biological samples, like saliva, often contain a diverse range of substances that could interfere with measurements, potentially leading to inaccuracies. Additionally, the precision of these biosensors is highly dependent on stable environmental conditions, such as consistent temperature and pH levels, meaning that they might require a controlled testing environment. Another noteworthy concern is electrode fouling, where substances from the samples may adhere to the sensor’s electrodes, impairing its performance and necessitating more maintenance, like frequent calibration. There are also practical hindrances, such as the sophistication and precision of the instrumentation necessary for accurate readings. This requirement can increase the costs and restrict the distribution and use of such biosensors, especially in low-resource settings. Ongoing advancements in sensor technology, materials science, and signal processing are crucial to overcome these challenges, ultimately facilitating wider adoption and more effective point-of-care testing in various medical environments. Table 6 briefly describes the advantages and disadvantages of electrochemical biosensors.

## 4. Challenges and Future Perspectives

The potential of non-invasive electrochemical biosensors for the detection of oral cancer is vast. This review paper explores the cutting-edge developments in electrochemical biosensors for early and accurate oral cancer diagnosis. While significant progress has been made, several challenges persist in this field. One of the main challenges is identifying specific and sensitive biomarkers that can reliably indicate the presence of oral cancer. Translating laboratory research into practical, user-friendly biosensing devices presents technical and engineering obstacles. The standardization of protocols and the validation of biosensors’ performance across different patient populations and clinical settings are crucial in establishing their clinical utility. Moreover, integrating electrochemical biosensors into the existing healthcare infrastructure requires collaborative efforts from researchers, clinicians, and regulatory authorities. However, the outlook remains promising, as advances in nanotechnology, biomarker discovery, and machine learning algorithms hold the potential to overcome these challenges. With continued research and innovation, electrochemical biosensors are poised to revolutionize oral cancer detection, enabling early intervention and personalized treatments and ultimately contributing to the improved management and prognosis of oral cancer patients. Amperometric, voltammetric, and impedimetric biosensors offer specific advantages and limitations in the context of oral cancer detection.

Amperometric biosensors offer the advantage of simplicity, easy on-chip integration, and potentially low costs and fast response times [140,141]. Their sensitivity and versatility enable the accurate and early detection of cancer biomarkers, contributing to improved diagnostic processes and treatment management, making them a promising tool for oral cancer and other malignancies [26]. However, challenges such as regular calibration and potential interference in the detection process should be carefully addressed for their effective use in complex samples, as in oral cancer detection [142]. Voltammetric biosensors have the advantages of simple on-chip integration and potentially low costs, serving as beneficial point-of-care diagnostic tools [140]. They can also provide comprehensive insights for the targeting and detection of cancer biomarkers. However, they require careful electrode design and corresponding materials to ensure the highly selective and sensitive detection of cancer biomarkers [143]. Impedimetric biosensors, on the other hand, possess the potential for the early detection and diagnosis of oral cancer, attributed to their high sensitivity, non-invasive nature, label-free detection methods, and capabilities for real-time monitoring [144]. On the other hand, they could be more valuable in other biomarker categories, where difficulties restricting their use are not present. Complex sample matrices and ambient conditions can also impact them, and they need precise devices to measure exact values. To summarize, impedimetric biosensors provide non-invasive and real-time monitoring capabilities, amperometric biosensors offer sensitivity and simplicity for oral cancer diagnosis, and voltammetric biosensors enable point-of-care diagnostics. Selecting a biomarker type for a given biomarker class is contingent upon the requirements for precision, user-friendliness, and environmental factors.

Furthermore, a range of studies have explored the potential of using machine learning and electronic tongues to discriminate between saliva samples from oral cancer patients and healthy individuals. Braz et al. demonstrated that supervised machine learning algorithms, especially support vector machines and random forest, achieved high accuracy in this discrimination [145]. Zlotogorski et al. similarly discovered that the Fourier transform-based spectrum of salivary exosomes and computer-aided discrimination analysis could be used to accurately distinguish between these two groups [146]. Kouznetsova et al. further expanded on this work by using saliva metabolites and machine learning to distinguish between oral cancer and periodontitis, achieving accuracy of 79.54% [147]. Lastly, Ishikawa et al. identified specific salivary metabolite biomarkers for oral cancer screening, which could be integrated into a non-invasive diagnostic method [50]. These studies collectively highlight the potential of machine learning and electronic tongues in improving the accuracy and efficiency of oral cancer diagnosis. In addition, future studies should also focus on recurring predictor biomarker candidates to reduce the mortality rates.

Modern AI systems implemented in image analysis and pattern recognition are attracting attention. They use deep learning algorithms to analyze clinical and microscopic images so as to identify subtle changes within malignant tissue. These algorithms are trained on vast datasets, potentially distinguishing between benign and malignant tissues. This will also be a valuable resource during clinical examinations so that healthcare providers can give a second opinion and monitor the patient’s health for better prospects. Smart devices and mobile health applications also show promising results for preliminary oral cancer screening [148,149]. These systems include either mouth safeguards or intraoral devices that are integrated with biomarkers, highly specialized cameras, and AI-powered technologies and can help to regularly monitor any potential risk [150]. Popovic et al. presented a platform that primarily addresses xerostomia via a personalized approach that involves a microfluidic chip embedded with a tooth model for targeted therapeutic delivery based on real-time oral cavity measurements [151]. Similarly, Yaduvanshi et al. proposed a machine learning-based approach that utilizes a modified local binary pattern (MLBP) to analyze oral lesions using histopathological images. The MLBP technique is employed for texture feature extraction, using deep convolutional neural networks to detect changes in the oral cavity due to cancer [152]. There is potential to extend this work by using other frameworks to identify diseased regions in oral cancer images. Deep learning (DL) is a subset of machine learning that simplifies complex data algorithms and helps to identify similar patterns; it could be used by healthcare professionals to monitor the conditions of patients. A notable increase has been observed in DL-based techniques for oral cancer diagnosis and prognostic prediction. Warin et al. discussed and reviewed the application of DL techniques by analyzing various DL models, focusing on the accuracy, sensitivity, and specificity across different imaging modalities [153]. The authors recommended the development of specific reporting protocols in a standardized fashion to improve the transparency and address issues like heterogeneity for improved diagnostic accuracy in DL applications. Hence, there is a need for future studies to implement standard methodologies to verify clinical images consisting of both cancerous and non-cancerous stages, so as to ensure the accuracy of the data while establishing the need for its usage in medical AI studies. This will provide a promising approach to improved DL design so as to potentially increase oral cancer patients’ survival rates. Interestingly, neural networks and particularly convolutional neural networks (CNNs) can help to enhance the accuracy of early detection by identifying subtle patterns indicative of early-stage lesions. By integrating genetic biomarkers, histopathological images, and clinical parameters with DL architectures, the development of predictive models for treatment response monitoring and prognosis is facilitated. Huang et al. utilized an oral cancer (lips and tongue) dataset consisting of 87 sets of cancerous and 44 sets of non-cancerous oral images [154]. They used a CNN-optimized combined algorithm to extract features from the images, removing noise while enhancing the visibility and diversity of the data. However, several challenges, such as limited and unequal sample sizes, can hinder such a model’s ability to create a generalized algorithm for future use. Building upon similar models, Lin et al. presented an effective smartphone-based oral cancer imaging diagnosis technique, using 688 lesion images and 760 normal mucosa images. A high-resolution network (HRNet-W18), an advanced DL model, was used to classify positive and negative cases of oral cancer, potentially creating a viable option for healthcare workers and patients in remote areas [155]. Thakuria et al. followed a similar approach to the application of DL techniques in the diagnosis of oral cancer with the use of smartphone and DSLR image analysis. The authors conducted a systematic review of 25 papers, highlighting several models for multiclass classification and object detection and focusing on parameters like precision, specificity, and accuracy [156]. However, a similar limitation could be observed regarding the need for larger and more diverse datasets from multiple healthcare facilities that can be integrated with lesion characteristics for improved diagnostic capabilities. As the research continues to evolve, there is immense potential for breakthroughs in oral cancer treatment via artificial intelligence-driven diagnostics and more advanced imaging techniques, leading to quicker and less invasive procedures. Moreover, the discovery of appropriate biomarkers could be helpful for the early diagnosis of oral cancer. This could eventually result in higher survival rates for patients.

## 5. Conclusions

This review delves into the various applications of biosensors with non-invasive diagnostics for oral cancer. Integrating biomarkers and biofluids into the development and application of biosensors enhances the potential for accurate, reliable, and non-invasive oral cancer detection methods. While each type of biosensor offers distinct advantages, further research and development are essential to address challenges such as optimization for clinical settings, the validation of real-world applications, and integration into established diagnostic pathways. Understanding these biosensors’ unique capabilities and limitations is crucial in harnessing their full potential in oral cancer detection, ultimately contributing to improved patient care and outcomes. As the field of electrochemical biosensing continues to advance, the strategic integration of these biosensors into clinical practice holds promise to transform the landscape of oral cancer detection and management.

## Figures and Tables

**Table 1:** Biomarkers used for the detection of oral cancer.

Biomarker	Candidate Biomarker	Sample Type	Type of Analysis	Sensitivity	References
	NOTCH1	Tissue	qRT-PCR	-	[56]
	CDKN2A	Tissue	NGS	-	[57]
	CASP8	Tissue	Whole-exome sequencing	-	[58]
	MMP9 (mRNA)	Blood/Serum	ELISA	NA	[59]
	VEGF (mRNA)	Serum	qRTPCR	NA	[60]
	MALAT1 (lncRNA)	Tissue	qRT-PCR	NA	[61]
	miRNA-21	Saliva	qRT-PCR	>65%	[62]
	miRNA-31	Saliva, Plasma, Serum	qRT-PCR	>70%	[63]
	miRNA-145	Saliva	qRT-PCR	>60%	[62]
	miRNA-196a and miRNA-196b	Tissue	qRT-PCR	>95%	[64]
Protein-Based Biomarkers	p53	Saliva Serum	ELISA	>87%	[65]
	Ki-67	Tissue	IHC	-	[66,67]
	EGFR	Saliva, Buccal cells	ELISA, RT-PCR	-	[68]
	EGFR	Tissue	Fluorescence molecular imaging	100%	[69]
	VEGF	Serum	ELISA	64%	[70]
	IL-6	Saliva, Tissue	ELISA, Western blotting, IHC	-	[71,72,73]
	IL-8	Saliva	ELISA, qRT-PCR	-	[74,75]
	TNF-α	Saliva	ELISA		[76,77]
	MMP9	Saliva	ELISA	-	[78]
	MMP1	Saliva	Immuno-MALDI	-	[79]
	CYFRA 21-1	Saliva, Serum	ELISA, qRT-PCR	93.8%, 88%	[80]

Abbreviations: qRT-PCR—quantitative reverse transcription polymerase chain reaction; IHC—immunohistochemical detection; ELISA—enzyme-linked immunosorbent assay; NGS—next-generation sequencing.

**Table 2:** Commercially available kits for early detection of oral cancer detection.

Test Kit Name and Location	Techniques Used	Sample Type	Dose/ Volume	Sensitivity	Specificity	Characteristics	References
OralCDx Brush Test Suffern, NY, USA	Brush biopsy combined with traditional cytopathology	Cells	No specific dose	Less than 4%	Less than 3%	Is non-painful, captures samples from all three layers of the epithelium, and shows accuracy in detecting pre-cancerous lesions	[87]
ViziLite Plus Phoenix, AZ, USA	Chemiluminescence using Toluidine Blue O (TBO) dye	Exfoliative cytology	No specific dose	100%	-	Helps dental professionals to detect oral cancer at early stages; it is effective for patients at increased risk of oral cancer, especially those with HPV infection or those over 40 years old	[88]
OralID Houston, TX, USA	Fluorescence visualization technique	Blood or tissue biopsies	No specific dose	96%	-	Helps to identify oral cancer and pre-cancerous lesions using fluorescence technology, highlighting abnormalities in the mouth	[89]
Oral Scan Pro Thiruvananthapuram, Kerala, India	Fluorescence imaging technology	Visual inspection	No sample required	83%	72%	Requires no direct biological samples like saliva or blood, enables early detection via a handheld multi-modal imaging system that captures real-time images of the oral tissue	[90]
Bio/Screen Oral Cancer Screening Kit Danbury, CT, USA	Fluorescence visualization technique	Visual inspection	No specific dose—oral mucosa	-	-	Functions as an adjunct tool for detection of potential abnormalities, highly portable	[91]
OraRisk HPV Test Brentwood, TN, USA	PCR	Saliva	No specific dose—oral mucosa	-	-	Specifically identifies HPV types and is recommended for patients with risk factors such as a history of oral cancer, sexually active individuals, and those with suspicious oral lesions	[86]
OncAlert Miami, FL, USA	ELISA	Oral mucosa	No specific dose	Under development	Under development	Identifies high-risk individuals	[92]
VIOME CancerDetect Bellevue, WA, USA	Meta-transcriptomics sequencing	Saliva	A few mL	90%	95%	It is non-invasive and employs RNA sequencing technology combined with AI for high accuracy	[93]
Galleri—Multicancer early detection test Menlo Park, CA, USA	Next-generation sequencing combined with methylation analysis	Blood	20 mL	Less than 1%	51.5%	Recommended for individuals at higher cancer risk, especially aged 50 and older	[94]
Quick Blue Oral Care Pvt Ltd. KIIT, India	Microscopic evaluation using Toluidine Blue O	Oral mucosa	No specific dose	93%	100%	Detects oral cancer cells in minutes and reduces the need for multiple biopsies. Particularly useful for cases of non-healing oral ulcers	[95]

**Table 6:** The advantages and disadvantages of electrochemical biosensors for the detection of oral cancer biomarkers.

Advantages	Disadvantages
High sensitivity with low levels of concentrations of cancer biomarkers	Interference from electroactive species in complex samples
Rapid response time	Batch-to-batch variability in fabricated sensors
Potential for miniaturization for point-of-care devices	Difficulty in integration into clinical practice
Real-time monitoring capabilities	Interfacial area degradation over time
No need for complex instrumentation	Accuracy can be affected by temperature and pH
Minimal sample preparation and consumption	Potential cross-reactivity and complex data interpretation
